# The chronic disease Self‐Management Programme: A phenomenological study for empowering vulnerable patients with chronic diseases included in the EFFICHRONIC project

**DOI:** 10.1111/hex.13430

**Published:** 2022-01-10

**Authors:** Dallal Fracso, Gérard Bourrel, Christian Jorgensen, Hélène Fanton, Hein Raat, Alberto Pilotto, Graham Baker, Marta M. Pisano, Rosanna Ferreira, Verushka Valsecchi, Yves‐Marie Pers, Agnes Oude Engberink

**Affiliations:** ^1^ IRMB, INSERM, CHU Montpellier University of Montpellier Montpellier France; ^2^ Department of Primary Care, School of Medicine University of Montpellier Montpellier France; ^3^ Institut Desbrest d'Epidémiologie et de Santé Public, UMR UA11 INSERM Université de Montpellier Montpellier France; ^4^ Department of Public Health Erasmus MC University Medical Center Rotterdam The Netherlands; ^5^ Department of Geriatric Care, Orthogeriatrics and Rehabilitation E.O. Galliera Hospital Genoa Italy; ^6^ Quality Institute for Self Management Education and Training Portsmouth UK; ^7^ General Direction of Care, Humanization and Social and Health Care, Ministry of Health Biosanitary Research Institute of the Principality of Asturias Asturias Spain

**Keywords:** chronic diseases, Chronic Disease Self‐Management Programme, empowerment, health education, phenomenology, vulnerable populations

## Abstract

**Introduction:**

The Chronic Disease Self‐Management Programme (CDSMP) has resulted in improved health outcomes for patients. However, research has focused mainly on those with chronic conditions and has not extensively explored prevention programmes targeting individuals with specific vulnerability profiles.

**Aim:**

This study aimed to understand the effects of the CDSMP on the lived experience of vulnerable patients included in the EFFICHRONIC project in France, based on their needs and expectations before and after participation.

**Methods:**

We conducted a qualitative phenomenological semio‐pragmatic study based on 37 in‐depth interviews with 20 patients (20 before/17 after CDSMP).

**Results:**

By transforming existential dimensions (identity, relationship with others and bodily experience), chronic illness generates new needs in the vulnerable person. By resonating with the expectations and needs of participants, the CDSMP induces motivation and a sense of belonging to a community of peers. It has enabled the participants to become actors of their own health *until empowerment*. Although some limitations are reported, the programme has awakened a desire in the participants to take better care of their health and to develop personal skills with, for some, a desire to become involved in health education.

**Conclusion:**

Our phenomenological approach highlighted the resonance between the programme (its design and implementation) and the lived experience of patients, as an effective element of empowerment. This necessitates training the facilitators to elicit the lived experience of patients. Furthermore, as a patient‐centred approach is required, the facilitators need to learn how to adapt the design of the programme to the singularity of the patient.

**Patient or Public Contribution:**

Patients provided the data that were collected through in‐depth interviews, and their experiences before and after the programme were analysed.

## 1 INTRODUCTION

The continuing increase in the number of people with chronic disease is one of the greatest challenges for healthcare systems worldwide. Chronic condition multimorbidity is high, with prevalence increasing with age (up to 50% after 65 years).^1^ The increasing proportion of older adults in the population, and of younger adults with chronic disease who will live to an advanced age, has huge consequences for policy and healthcare expenditures.^2^ It has been estimated that chronic diseases may account for nearly 60% of overall mortality.^3^ In most countries, the poorest people are at the highest risk of developing chronic disease.^4^ The authors have added that vulnerable populations are of more concern because chronic diseases are responsible for 50% of the total disease burden in low‐income countries.^4^

Vulnerable populations refer to a wide range of groups, including the economically deprived, the ethnic minorities, the elderly or those who encounter barriers to accessing health care (geographical isolation, low access to public transportation, lack of social networks).^5^, ^6^ Several studies have reported almost an additional 10% of chronic disease in vulnerable populations.^7^ They state that patients living in poverty are much more likely to be in poor health and to have disabling conditions, and are less likely to have used many different types of health care. Vulnerability may thus promote the incidence of chronic disease due to a higher frequency of risk factors in this population. Conversely, vulnerability can also be the consequence of chronic disease.^8^ The work of Jeon et al.^9^ demonstrated how socioeconomic and cultural differences produce short‐ and long‐term inequalities in terms of self‐care and health. This supports studies showing that chronic illness aggravates social vulnerability or precipitates people into precarity through loss of work.^10^

A growing number of behavioural interventions to help people live with chronic diseases are emerging.^11^ Among them, self‐management involves actions and behaviours necessary for the protection and promotion of health and for the management of the physical, emotional and social effects of the chronic disease. In a study of diabetic patients with vulnerability criteria (limited spoken English, uninsured and/or poor school education), a supervized, structured self‐management programme demonstrated better outcomes than in usual care.^12^ However, in the same population, a recent study^13^ identified barriers limiting the implementation of self‐management (difficulty in accessing care/healthcare, interference with the social environment, lack of motivation). In addition, older people with low incomes consider health care and healthy ageing less essential than a population of the same age with higher incomes.^14^ On the other hand, three factors would favour the benefit of self‐management in this population: peer support, a patient‐centred approach and positive empowerment. Vulnerable people participated less often in self‐management programmes and differed in support preferences (identifying community resources, improving communication and shared decision‐making with healthcare providers).^15^ Taken together, vulnerable people represent patients with the greatest need for education and support, but they are characterized by the highest task frequency and lowest self‐efficacy, resulting in weaker adherence to self‐management programmes. These findings may lead to a better tailoring of self‐management approaches to address the specific requirements of the vulnerable population with chronic diseases.^16^ Among the self‐management techniques, the Chronic Disease Self‐Management Programme (CDSMP)^17^ was developed by a research group at Stanford University (US), and was built based on the qualitative work of Corbin and Strauss.^18^, ^19^ Since then, the CDSMP has been successfully implemented face to face around the world.^20^, ^21^, ^22^ It includes the development of an action plan, and provides feedback with respect to problem‐solving skills and other desired behaviours. It also serves as a guidance and training in attaining competencies such as the reinterpretation of symptoms and disease management. The conceptual elements underlying the methods of the CDSMP are self‐efficacy, empowerment, peer‐to‐peer education, recognition of the social determinants of health, community participation and risk stratification.^23^ A few studies have recently been published and have highlighted a positive benefit of the CDSMP regarding ethnic minorities or low‐wage workers.^24^, ^25^, ^26^, ^27^

Recently, the EFFICHRONIC project (http://effichronic.eu/) aimed to evaluate the CDSMP intervention in five European countries (France, Italy, Netherlands, United Kingdom and Spain) by specifically including adults with a chronic disease and vulnerable conditions (low income, social or geographic isolation) as well as their caregivers.^28^ The revised Medical Research Council guidelines^29^ have emphasized the need not only to measure outcomes but also to detail the process of implementing the intervention under study. Michie et al.^30^ argued that qualitative or mixed methods can be used to understand health behaviours and thus identify effective components of a complex intervention such as the CDSMP. Although the CDSMP improves health, little is known about how participants experience the programme or the processes involved.

In this article, we conducted a qualitative study with vulnerable patients included in the CDSMP of the EFFICHRONIC project in France. The aim was to understand the effects of this programme by collecting information regarding the patients' lived experience, needs and expectations before and after their participation. This knowledge could help us to better understand what works in a complex intervention and to integrate it into educational strategies.

## 2 MATERIALS AND METHODS

### 2.1 EFFICHRONIC project intervention

The CDSMP consisted of a series of six workshops, 2.5 h each, which were held once a week for 6 weeks. The number of participants was no more than 15. An important conceptual element of the intervention concerned peer education. Participants with a chronic condition were encouraged towards self‐management by professionals as well as peers (other participants with a chronic condition). One professional and one peer would volunteer together to lead a series of workshops. To this end, professionals and peers were recruited and trained on the CDSMP principles. Generally, each workshop had between seven and eight activities, with specific objectives to achieve. Each one integrated different techniques (individual work, small and large groups, brainstorming, etc.), had an agenda and time defined for each activity. Over the course of six workshops, participants were able to (i) set realistic health goals, (ii) learn to self‐manage pain and discomfort, (iii) learn to self‐manage their diet and (iv) learn to self‐manage physical activity, mood and the way the disease influenced their personal relationships. The intervention is designed to actively involve participants. Through different activities, participants are supported to make different changes that increase confidence in their ability to manage the disease. The facilitators create the climate appropriate for changes to occur. For this, it was important not to judge people or their actions, to respect confidentiality in the workshops and to support fair participation.

### 2.2 Design of the qualitative study

A qualitative semio‐pragmatic phenomenological study was chosen to describe and understand the lived experience of vulnerable and chronically ill patients before and after their participation in the CDSMP intervention of the effichronic project.

This report was guided by the COnsolidated criteria for REporting Qualitative research (COREQ).^31^

### 2.3 Participants and recruitment procedures: Purposive sampling

All inclusion and noninclusion criteria of the EFFICHRONIC project have been described elsewhere.^28^ Briefly, conditions of vulnerability included older people (over 65 years) living alone or in retirement homes, or in a situation of social or family isolation; persons receiving a disability pension or allowance; low‐income ethnic minorities; and low‐income legal immigrants, refugees and/or asylum seekers. For asylum seekers, their residence had to have been known for at least 6 months. Low income was defined as below the poverty line at 60% of the median standard of living for the year 2015.^32^ To evaluate precarity, we used Gijon's social‐familial evaluation scale, which takes into account the person's familial and socioeconomic situation, housing, social relations and social support and assistance. The score is interpreted as follows: between 10 and 14: vulnerability in social support; 15 or greater: important lack of social support.^33^

Our qualitative study was presented to the EFFICHRONIC leaders and facilitators orally, followed by an email that was then sent. Participants in their groups were asked to partake in the study. The contact details of the volunteers were given to the investigator, who then contacted them by telephone to agree on a date and place for the first interview before their participation in the programme. They knew they would be contacted 6 months later to arrange the second interview after their participation in the CDSMP. The location of the interviews was left to the convenience of the participants. Fifteen of the first interviews were conducted at the participants' home (then 13 interviews at a 6‐month interval). This had the advantage of placing the person in his/her living environment. Four interviews took place in rooms in the administrative centre of the City A University Hospital. One interview took place at the ‘Secours Populaire’ (a non‐profit French association engaged in providing food aid, clothing, access to and maintenance of housing, access to health care, socioprofessional integration, access to cultural activities and, more generally, access to rights for all) of City A. The interviews were carried out in urban, semi‐rural and rural areas, and reflected a range of occupations and income levels of participants.

As qualitative research is an iterative process of sampling; required sample sizes cannot be calculated a priori. We used purposive sampling to obtain a diversity of patients' experiences.

The following criteria guided participant recruitment: age, sex, speaking French, educational level, vulnerability conditions and the experience of living with a chronic disease.

### 2.4 Data collection: Semi‐structured in‐depth interviews before and after participating in the CDSMP

The interview guides (Supporting Information 1 and 2) were constructed with phenomenological questioning to allow for a reflective process. They were designed with the help of two researchers in qualitative methodologies. They explored different dimensions of the studied phenomenon: the impact of a chronic disease on the patient's life (diagnosis, representation, experience, difficulties, health determinants, relationship with others, etc.); expectations of the health educational programme (course, topics, motivations); and the changes experienced after their participation in the programme. The methodology team checked the appropriateness, as well as the intelligibility, of the questions in two initial test sessions administered by the investigator. Follow‐up prompts were designed to encourage participants to recount their personal experiences as authentically as possible. The investigator received preliminary training on phenomenological reformulation (prompts) to carry out the in‐depth interviews. She reported her involvement after each interview.

After reading the briefing note, the investigator introduced herself as a junior general practice doctor (junior GP) working on her thesis. Before the interview began, the research objectives of the interview were explained to the participants. She made sure to create an atmosphere of confidence such that answers would be spontaneous and truthful. The respondents were informed that their responses would remain confidential and anonymized, meaning that their personal identification details would not appear. They were made aware of the possibility of being able to stop the interview at any time without any reason, and to withdraw from the study if they wished. The interviews were recorded. The sound quality was sufficient to produce audible and understandable voice files. The recordings were transcribed verbatim. We did not plan to collect nonverbal data. The interviews were anonymized by a coding from A to T (the letter A corresponds to the first participant interview and T to the twentieth) associated with number 1 for the interview conducted before the programme, and number 2 for the interview at 6 months.

### 2.5 Analysis

Pragmatic phenomenology is a descriptive method of categorizing lived experiences recorded in interview transcripts. In this semio‐pragmatic method,^34^, ^35^ the analyst considers all the semiotic elements of a text, including linguistic and contextual clues. The approach of this semio‐pragmatic analysis (Table 1) is based on several processes: on the semiotic characterisation of the selected themes, on a process of constant comparison^36^ to construct empirical categories and on a principle of logical data ordering inspired by C. S. Peirce's theories.^37^ As a result of this ordering, the category that is the most conceptually dense (i.e., the highest level in the hierarchical classification of signs) controls the meaning of the phenomenon at stake.

**Table 1:** Semio‐pragmatic analysis steps

Word‐for‐word transcription of recordings (French: verbatim)
Reading using a floating attention, followed by a focused reading
Dividing text into meaningful sections and assigning a theme to each section
Identifying all textual and contextual clues that are relevant to the research question
Proceeding to a semiotic characterisation of these themes according to the theory of signs of C. S. Peirce
Assembling and ordering these semiotic elements to construct first‐level empirical categories
Raising these categories into a more general category by constant comparison up to theoretical saturation
Modelling by putting the main results in order in a comprehensive synthetic statement

The last step consists of restoring the meaning of the studied phenomenon in all its dimensions in the form of a general synthetic statement. Investigator triangulation was achieved by compiling the analyses of the two qualitative research experts and of the trained investigator. The interviews were stopped upon reaching data saturation, without the need to add more participants.

### 2.6 Research ethics and patient consent

The protocol was approved by the French ethics committee (CPP SOOM I − 2018‐A01054‐51). The study was registered at ClinicalTrials.gov (NCT03840447) and was conducted in accordance with the Helsinki declaration. Participants provided written consent for publication before enrolment.

## 3 RESULTS

### 3.1 Participant characteristics and interview descriptions

Theoretical saturation was reached after including 20 participants. All interviews were performed by the same person between 4 March 2019 and 7 September 2019.

The first 20 interviews (on the impact of the disease and the expectations of the programme) were conducted before the first CDSMP workshop (15 days before at the earliest, and the day before at the latest). They lasted approximately 40 min (minimum 20 min and maximum 55 min). Among the participants, 15 were of French origin, 5 were from the Maghreb area of Africa (Algeria, Morocco and Tunisia) and one was a member of the Roma community. French was their usual language.

Seventeen of them participated in the second interview (focusing on the experience of the programme and the changes identified after the programme), conducted 6 months later. One person did not participate in the programme, and two could only follow part of the first session. At her suggestion, the first person participated in an adapted physical activity programme. She was not able to do both, but still wished to participate. The other two felt that it would be too difficult for them emotionally, after participating in the first 30 min of the first CDSMP session. They preferred not to continue. The other 17 participants attended the required number of sessions to complete the training (at least three workshops). The second interview lasted for an average of 17 min (minimum 10 min, maximum 25 min). See Table 2 for the participant characteristics.

**Table 2:** Participant characteristics

Patient	Age/gender	Zone^a^	Occupation/monthly income	Marital status	Diseases	Disease acceptance status**	Education level	Precarity score***	Workshop sessions range 0–6
A	60/F	A	No professional activity/disability payment (DP)/not specified	Couple	Rheumatic	Accepted	First 2 years of undergraduate degree	10	6
B	33/M	A	No professional activity/DP/1730€	Single	Neuromuscular	Accepted	Undergraduate degree	14	4
C	47/M	A	No professional activity/DP/1730€	Couple	Neuromuscular	Accepted	High school education	8	5
D	71/F	A	Disability retirement payment/1730€	Couple	Rheumatic	Accepted	First 2 years of undergraduate degree	6	5
E	63/F	A	Disability retirement payment/1730€	Couple with children	Rheumatic	Depressed	Postgraduate degree—Masters	8	6
F	47/F	A	Executive/DP/between 1154€ and 1730€	Single	Rheumatic	Depressed	Postgraduate degree—Masters	8	6
G	57/F	A	No professional activity/DP/1730€	Couple	Neuromuscular	Accepted	Baccalaureate/technical diplomas	10	6
H	44/M	A	No professional activity/DP/1730€	Couple with children	Neuromuscular	Angry	Baccalaureate/technical diplomas	9	5
I	22/M	A	No professional activity/DP/1730€	Single	Neuromuscular	Accepted	Baccalaureate/technical diplomas	13	3
J	66/F	C	Retired/between 635€ and 1154€	Single	Respiratory	Depressed	Baccalaureate/technical diplomas	9	6
K	75/F	C	Retired/between 1154 and 1730€	Single	Rheumatic	Accepted	Postgraduate degree—PhD	12	0
L	56/F	C	No professional activity/RSA^a^	Single with children	Irritable bowel syndrome	Denied	High school education	12	1
M	44/F	C	No professional activity/RSA	Single with children	Neuromuscular	Accepted	Baccalaureate/technical diplomas	12	6
N	53/F	C	No professional activity/DP/between 635€ and 1154€	Single with children	Neuromuscular	Depressed	Baccalaureate/technical diplomas	15	6
O	54/F	B	No professional activity/RSA	Single with children	Respiratory	Denied	Baccalaureate/technical diplomas	14	6
P	60/F	B	No professional activity/RSA	Single	Rheumatic	Accepted	Baccalaureate/technical diplomas	10	5
Q	50/F	B	No professional activity/RSA	Couple with children	Depression, stabilized	Depressed	Primary school education	11	6
R	55/F	B	No professional activity/RSA	Single	Neuromuscular	Depressed	High school education	14	6
S	49/F	B	No professional activity/RSA	Couple	Depression, stabilized	Depressed	No schooling	14	1
T	52/M	B	No professional activity/RSA	Single	Rheumatic and respiratory	Angry	Baccalaureate/technical diplomas	14	5

^a^ Zone A is urban, B rural, C semi‐rural.

** psychological stages of serious illness according to E. Kübler‐Ross.

*** Gijón Scale : It takes into account the family situation, economic, housing, social relations, and socio‐human support of the person. Between 5 and 9: the social situation is good or acceptable. Between 10 and 14 the person is socially at risk. Greater than or equal to 15 there is a social problem.

### 3.2 Findings of the semio‐pragmatic analysis

Our analysis highlights the logical emergence of four phenomenological categories (also named the phenomenological statement) and an analytical figure that allows us to understand the three conditions at play in an intervention to guide towards empowerment.

#### 3.2.1 By transforming existential dimensions (identity, relationship with others and bodily experience), chronic illness generates new needs in the vulnerable person

Participants report a sense of rupture with their life before the chronic illness. First, they experience a loss of self‐esteem, a loss of ability and a sense of being diminished: ‘Being aware that everything is over, a loss of all capacities, it's hard on the self‐esteem to say that I'm diminished’ (F1). ‘My whole life has been turned upside down. You know, all the time you haven't mourned for your life before, then you can't move forward’ (G1). Because of the stigma of chronic illness, they feel socially excluded. ‘In fact, people shun you because you represent the disease’ (M1); ‘I wonder why I'm constantly being rejected’ (R1).

This generates a need for recognition and communication with those around them, which, for professionals, requires a patient‐centred approach: ‘the worst thing is not being acknowledged’ (R1), ‘I have a good medical team that listens, that talks with you, it's very important’ (S1) or ‘I'm lucky to have the support of my husband and my daughter’ (A1).

Second, the social vulnerability of people with chronic illness is responsible for a loss of social and financial autonomy associated with job loss: ‘What bothers me the most is no longer being able to work, because you're excluded from society’ (N1, D1); ‘it's the financial side that takes over’ (M1). It is a circular process: chronic illness makes it difficult to access social support, and exacerbates the disability. This vulnerability is aggravated by the difficulty of accessing care, the lack of understanding of health professionals and the lack of adequate information about the disease, which confuses the care pathway: ‘No, we've been told to go to the hospital. How do I do this if I have no gas in my car?’ (O1); ‘the hospital environment, medical things are hard, and we don't understand anything. Doctors use terms that we don't really understand’ (M1); ‘We need a center where we can have information, ask questions’ (R1). Their need of information concerns ‘all the rights we can benefit from and how to get them, social assistance, the organizations that exist’ (H1, 1I).

Finally, all these difficulties have a serious impact on the bodily experience of chronic illness, on the experience of physical and psychological pain and on anxiety about the future: ‘a real torture, when you can't fall asleep at night, the next morning you are no longer a human being’ (Q1). ‘It's also stressful because we wonder what we need to do if something happens to us’ (J1). ‘I feel really fragile, I'm always afraid’ (E1).

#### 3.2.2 By resonating with the expectations and needs of the participants, the CDSMP induces motivation and a sense of belonging to a community of peers

We found that the design of the programme corresponds to the expectations and needs of the participants, which is a source of motivation. The first component is the location of close proximity, a familiar and nonmedical setting: ‘I know the place, so it reassures me’ (G2, S2), ‘discreet and very well located’ (R2). The second is a need for a patient‐centred approach with personalized guidance and support: ‘organizing therapeutic education programs without taking into account the way the patient sees it is not adapted’ (F1). Finally, participants emphasize ‘the enormous importance of the group in an education programme and the goals we set for ourselves that allow us to do what we wouldn't do’ (F2). The group allows them to share experiences, ‘to be heard and understood’ and ‘to overcome their fears’ (J1). It helps them to better understand their disease, their rights and how to secure them: ‘we know we're not alone. Sometimes it's easier to talk to other people who are experiencing the same problem’ (A1, B1, D1, M1, Q1). However, design is not enough. Implementation factors contributed to the success of the project by creating a sense of community. Participants need a structured programme with clear objectives, adapted to their individual pace and implemented by empathetic facilitators. The medical setting was converted from an educational workshop with ‘a friendly atmosphere where we have coffee, and a laugh’ (C2) that gives confidence and ‘the desire to go with enthusiasm’ (J2), with ‘the feeling of being at school’ (T2). The interactive method with learning objectives and feedback was interesting for them, as A2 said: ‘the development of an action plan and the feedback were very positive’. They all appreciated the patient–healthcare professional pairing that they found ‘very good, human and very pedagogical’ (E2).

Together, these factors provide them with a sense of belonging to a community of peers going through the same experience, and this breaks the isolation: ‘I realized it was as hard for others as it was for me… you no longer feel alone. And that's enormous’ (F2, H2, P2). ‘It was more than anything being in a group, not feeling alone with this illness’ (I2, M2, O2).

#### 3.2.3 The CDSMP made the participants actors of their own health until they became empowered, by changing their lived experience

By restoring the patients' negative experiences, such as loss of self‐esteem, feelings of social exclusion and physical fragility, participants felt ‘a renewed sense of self‐confidence and pride’ (B2, C2, Q2, J2) that empowered them to act differently. The programme enabled modifications to their lived embodiment with ‘renewed vitality’ and ‘positive thoughts’: ‘I feel like I'm living again’, said Q2. Their bodily experience is also improved with less pain and anxiety: ‘So it actually worked, as the pain went down, the stress went down’ (F2). It changed relationships with others with greater openness: ‘It helped me talk to my family and get their support’ (A2), and ‘I made friends’ (G2*)*. ‘You feel more free, more equal to others. It simplifies the relationship with the caregiver’ (E2). In fact, by regaining energy and a sense of self‐efficacy, they become actors of their own health: ‘they were able to put things in place and stick to them’ (F2). For this person, it is the group that allows that ‘the experience of others leads to a better self‐care’. Finally, some participants have started to ‘have goals that they didn't have at all’ (M2), and they are able to methodically plan their actions and to establish ‘the list of tasks, to better carry out a project, to finish it’ (B2) in complete autonomy.

Overall, the programme improved their knowledge and understanding. They learned the importance of nonpharmacological interventions, which was one of their expectations: ‘I'm waiting to see if there are more gentle methods without drugs’ (L1) such as ‘physical activity, nutrition, psychotherapy, relaxation’ (O2). For them, these methods can ‘delay the progression of the disease’ (F1), and contribute to self‐management and support: ‘it has changed my own care; I bought myself an exercise bike that I use regularly; I do physio in the swimming pool, it's excellent—I do it every other day’ (M2). These transformations have changed their relationship to health and have shown them that ‘one can live well with one's illness’ (E2, F2).

#### 3.2.4 Although some limitations are reported, the programme has awakened a desire to take better care of one's health and to develop personal skills with, for some, an enthusiasm to become involved in health education

Barriers to empowerment related to the programme that emerged in the study included the degree of acceptance of the illness, social isolation, lack of follow‐up and too short a period of support.

Some were unable to sustain their actions or maintain their goals. Patients emphasized their desire to be supported, to have the opportunity to revisit or expand on certain ideas and concepts, to continue beyond the programme, to go into more detail, as a sign of their commitment: ‘perhaps to go into more depth on the medical aspects, on the contact with doctors, as sometimes it's not very clear’ (C2). The lack of personalized support over the long‐term personal support is the main limitation attributed to the programme. This is the case for M2, who finds it ‘*too short’*. Thus, this person feels the need for additional information, ‘to go deeper into the medical aspects, depression, psychological aspects’, or the care pathway that remains unclear for H2: ‘when I say care pathway, it's also all the other health aids you can have on your side’. E and M would have needed ‘final individual debriefings’ with health professionals. Some said they needed guidance, and suggested continuing with a session every two to three months to maintain follow‐up and effort. ‘To do it all the time, to go deeper… I need someone to guide me’ (M2, F2).

However, what seems most important is the realisation that knowledge can be found within the group and in the exchange of experiences between peers: ‘Finally, it's between us that we exchange our stuff, we're the ones who really know’ said F2, who added ‘we'll try to see each other outside, to continue our mutual aid’. The group enhances individual commitment: ‘I did things that I never thought I would do, because there were commitments, but for the whole group it was like that’ (E2). *A2* continued to be involved: ‘I put action plans in place that I still carry out today’. F2: ‘it also helped me to tell myself to change my treatment, so I'm going to switch to immunosuppressants. My rheumatologist has been trying for 13 years’. O2 decided to get dentures, change his glasses and quit smoking.

**Figure 1:**
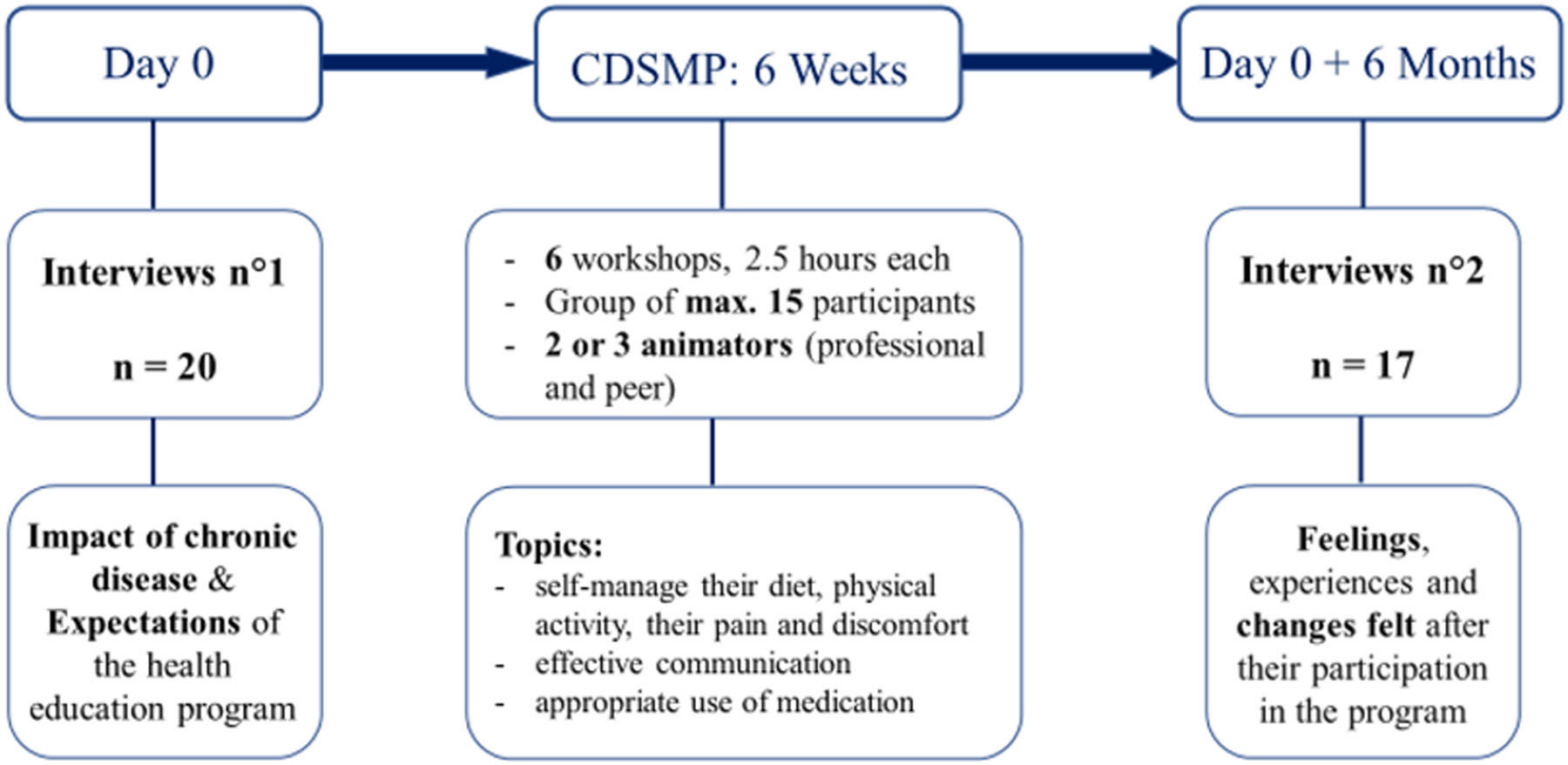
Study process. CDSMP, Chronic Disease Self‐Management Programme

For most of them, the programme allowed ‘a collective awareness of many things’ (F2), and revealed the importance of therapeutic education: ‘it's really something to develop for us, for our well‐being and to spend less time in hospital’ (G2); ‘I met other people for whom therapeutic education changed their lives’ (F2). This gave him ‘the desire to go further’ and even to enrol in a university patient‐expert diploma. Similarly, C2 stated: ‘my goal is to continue doing this, it's one of my plans to share my experience with them’ (Figure 1).

#### 3.2.5 Analytical figure: The three conditions leading to empowerment

**Figure 2:**
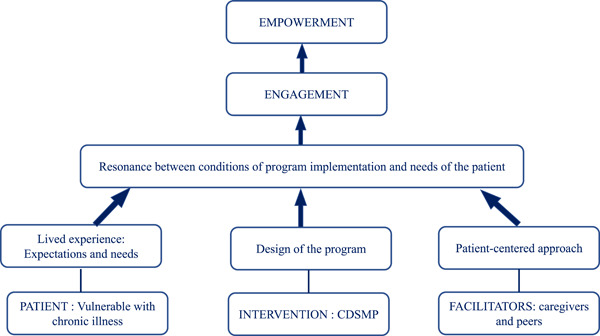
The three conditions leading to empowerment. CDSMP, Chronic Disease Self‐Management Programme

Figure 2 presents the three conditions of the process set in play in the empowerment of vulnerable patients with chronic illness, by the CDSMP.

1. *Patients* able to convey their experience during an interview and whose needs correspond to the elements of the programme design.
2. A *design* that responds to the needs of the chronically ill patients: The CDSMP was designed to operate within a framework of support, to provide appropriate information, community support, a reinforcement of personal efficacy and to enable empowerment.
3. *Facilitators*, caregivers and peers, capable of eliciting the lived experience, expectations and needs of patients with chronic illness, and of implementing the programme in a way that is adapted as closely as possible to these needs, through a patient‐centred approach.

## 4 DISCUSSION

Our findings demonstrate that the vulnerable patient with a chronic disease (i) has a specific experience, (ii) encounters difficulties in understanding the whole process of care and (iii) finds it difficult to access the different health professionals and social assistance. All of these issues generate needs. Through our study, we have shown that certain characteristics of the CDSMP were able to meet the needs of the participants by transforming their lived experience. This in turn induced motivation along with a sense of belonging to a community of peers, and promoted empowerment.

We have shown the importance of identifying barriers and facilitators through participation in a self‐management programme, particularly among the vulnerable population.

The first category showed that chronic illness transforms the existential dimensions of experience. The participants reported a feeling of rupture in their lives before and after the onset of illness, with repercussions for dimensions of their lived experience in a way that is defined by Heidegger^38^: identity (self‐esteem, feelings of being diminished),^39^ the relationship with others (stigmatisation, social exclusion),^40^ relating to their body and its image (physical and mental disorders, disability)^41^ or to time and the ability to make plans.^42^ Self‐esteem and relations with others are the two dimensions most profoundly impacted by chronic illness, having effects on personal and social vulnerability. This reinforces previous work on self‐efficacy,^43^ or on ‘renegotiating the self’ (like in Donnelly's meta‐analyses)^44^ or on social isolation.^45^, ^46^ Participants related a painful physical and psychological experience of chronic illness rendering them fragile up to the point of feeling that they were no longer ‘a human being’. They needed to understand their illness, starting with knowledge appropriate to them and their situation, and to develop self‐care skills. This accords with the findings of Mackey et al.,^47^ but, above all, with receiving recognition and improving communication with others. Morris^42^ has studied the phenomenology of chronic illness. He shows how a chronic illness can, in lived experience, manifest itself as a disruption of our usual relationship to bodily temporality, and thus as a disruption of freedom. Making an ‘existential diagnosis’, and allowing the patient to verbalize these new needs, seems important before the implementation of a programme. From this perspective, Marsh et al.^48^ and Burt et al.^49^ also demonstrated the importance of collecting patient experience as a source of authentic information for caregivers.

The second and third categories highlight that when there is a resonance between the lived experience of vulnerable patients with chronic disease (their expectations and needs) and the design of a programme, it enables motivation, a sense of belonging and engagement of participants within empowerment. Indeed, a positive transformation of the experience was observed: ‘loss of self‐esteem’ was replaced by ‘pride’ and ‘a sense of self‐efficacy’, social exclusion by ‘a sense of belonging to a community of peers’, the feeling of being diminished by the perception of ‘vitality’ and the lack of knowledge by ‘a need to learn’. Actually, this ‘community of peers’ created a break in social isolation, enabled a reconstruction of personal identity, built confidence and encouraged an understanding of the other, with a feeling of usefulness along the principles of peer support, as in Merdsoy's study^50^ of Canadian homeless people.

Our study also highlighted the role of access to information and understanding of the healthcare system in chronic illness. This is consistent with Mackey's work,^47^ showing that low literacy can lead to chronic illness. However, the study also showed that the adapted design is not enough. Favourable implementation conditions are needed. Among them, participants indicated that a person‐centred approach by facilitators is essential for change. This reinforces the work of Michie et al.,^30^ who have shown that, for behaviour change to occur, it is necessary to have what they call ‘active ingredients’ of implementation (*what* works, *why* it works and *how* it works). Our work has brought out a list of these facilitating factors: the reassuring place, the environmental support (the convivial nonmedicalized setting), the design of the programme in adequacy with the needs, the social support (belonging to a group, to a community of peers), the personalized coaching, the competences of the caregivers (empathy, listening, their pedagogical quality), the motivation and the commitment of the actors and the learning framework.^51^ Working within a framework emphasizes the resolution of problems to develop individual solutions through collaborative processes and reinforces the capability of each individual to find the means needed to acquire a healthier way of living (motivation, proactive behaviour, nutrition, physical activity).^23^

The last category shows that the programme raises awareness of the importance of health education and awakens a desire to take better care of one's health and to commit to developing skills. This strong commitment leads to becoming an actor of one's own health. Participants stated that ‘sharing the experience of others leads to better self‐management’. They emphasized the ‘enormous support of the group’ as the main determinant of empowerment, which is the goal of any educational process. These findings help us understand the mechanisms and processes that come into play using an approach that is one of empowerment,^52^ as illustrated in Figure 2. Following Aujoulat et al.,^53^ ‘the empowerment of the patient does not signify only the management of his or her treatment and the participation in decisions regarding his or her health. It acts as a personal transformation, concerning their identity, which culminates in a feeling of security, an acceptance of his or her body‐image, a sense of control, when the exigencies of the illness are integrated into a reconciliation with self’.

Finally, our work has shown that some participants still need support and follow‐up, while others show an enthusiasm to make a lasting commitment to collective health promotion. These are patients who have become aware that ‘therapeutic education has changed their lives’, that ‘sharing experiences with peers is essential’. This means that, alongside scientific knowledge, there is ‘experiential lay knowledge’ that must be taken into account and that can be helpful to their peers.^54^ This is what leads to autonomy and human dignity.

In a programme that is not focused on any one specific disease, it would be useful to offer a final interview to each person to identify their needs: those who need to consolidate some of their skills and direct those who could become patient‐experts to appropriate training. Some have not been successful in maintaining their actions or in sustaining their goals, due to a lack of personalized support. Various perspectives have been proposed to extend the effects of an intervention. Chen^55^ and Guell^56^ have shown that telephone support can improve certain dimensions. In North America, research by Kelly et al.^57^ on the intervention of peer *navigators* emphasized their effectiveness on the use of primary care, reducing the consumption of medications for psychiatric patients and increasing the feeling of self‐efficacy and empowerment.

### 4.1 Strengths and limitations of the study

This study hypothesized an internal methodological consistency between the researched object (lived experience), the phenomenological approach to data collection and the semio‐pragmatic data analysis, promoting a logic of emergence. One of the strengths of the study is that it has analysed, in the same population, the experiences and expectations of a health education programme before and about 6 months after participation. Semio‐pragmatic analysis allows precision of the logical constructs of a studied phenomenon by using Peirce's Theory of signs, limiting investigator‐related interpretation bias. Moreover, the participants were suffering from different diseases, and had a number of different demographic characteristics (age, sex, origin, family situation, different socioeconomic situations, living places). The methods used made it possible to highlight commonalities in the patients' experiences, despite these differences.

The qualitative phenomenological approach privileges a form of questioning that allowed us to access the richness of their lived experience of chronic illness in a way that is patient‐centred.^58^, ^59^

Conversely, there was a male/female imbalance in our population that can be considered as a limitation, although this was representative of the population participating in the EFFICHRONIC programme (75% women). We should also acknowledge a potential selection bias in the programme, some people having already participated in therapeutic education workshops. This profile of patients may be more invested in their chronic illness, but we could suppose them also to be less receptive to a novel educative intervention. On the other hand, the majority of the participants included in our study were selected by the facilitators (bias towards social desirability). However, the process of empowerment makes it possible to consider that if the participant had initially accepted to partake in the context of what he or she knew of the facilitator, the transformation of his or her lived experience could not be attributed to that facilitator.

## 5 CONCLUSION

To the best of our knowledge, this is the first qualitative study to evaluate the CDSMP with pre‐ and postinterviews. Our phenomenological approach highlighted the resonance between the programme (its design and implementation) and the lived experience of patients, as an effective element of empowerment. This necessitated training the facilitators to elicit the lived experience of patients, and, as a patient‐centred approach is required, facilitators had to learn to adapt the design of the programme to the singularity of the patient. Despite some limitations, the programme has awakened a desire in the participants to take better care of their health and to develop personal skills, with some wanting to become more involved in community health education. Personalized follow‐up actions complementing this type of intervention might provide an additional benefit for participants.

## CONFLICT OF INTERESTS

The authors declare no conflicts of interest.

## AUTHOR CONTRIBUTIONS

Yves‐Marie Pers, Christian Jorgensen, Hein Raat, Alberto Pilotto, Graham Baker, Marta M. Pisano, Rosanna Ferreira and Verushka Valsecchi participated in the implementation of the effichronic project. Agnes O. Engberink, Dallal Fracso, Hélène Fanton, Gérard Bourrel and Yves‐Marie Pers developed the qualitative study protocol. Dallal Fracso conducted the data collection. Agnes O. Engberink, Dallal Fracso and Gérard Bourrel analysed the data to saturation. All authors contributed to the writing of the article.

## Supporting information

Supporting information.

## Data Availability

The data are available upon request, with agreement from the EFFICHRONIC consortium. The data will be provided to researchers having a protocol for analysis. For further information, contact the corresponding author.
