# Supplementary material for: The chronic disease Self‐Management Programme: A phenomenological study for empowering vulnerable patients with chronic diseases included in the EFFICHRONIC project
Source: Health Expect. 2022 Jan 10;25(3):947–58. doi: 10.1111/hex.13430 (PMC9122418; doi:10.1111/hex.13430)
Supplement: Supplementary file 1 — Supporting information. [file HEX-25--s001.docx]

**SuPPORTING INFORMATION**

SuPPORTING INFORMATION 1: Interview guide before CDSMP

**Characteristics of the person:** First of all, some questions to better get to know you:

- Do you have a daily activity? *(profession, association, childcare, hobbies…)* ?
- Do you live alone, or with another person, or other people?
- How did you come to know about the program? Who referred you to us?

**Characteristics of the illness:**

- Tell me about your illness?
- What has changed since your diagnosis (diagnoses)?

**Factors determining health:**

- *Social environment*: How would you describe the impact of your way of life and your work on your health?
- *Characteristics of daily life:* Can you describe aspects of your daily life such as (physical activity, diet, quality of sleep)

**The health education program:**

- How would you like the program to unfold?
- What do you think of the fact that it will take place in a group of several patients?
- Can you describe to me the reasons why you’re participating in the program?
- What would you like to see in the program? What are your expectations for the workshops?
- What would you wish to change in your life to improve your health?
- That is all the questions I have, is there anything you’d like to add? Thank you very much for your time.

SuPPORTING INFORMATION 2: interview guide following the CDSMP

***Interview guide for feelings and motivations (6 months after)***

The questions listed here are not necessarily posed in exactly this manner, but the global theme will be presented, according to the interaction; the interviewer will adapt to the interviewee. The information in italics is indicative for the interviewer to be used as a stimulus in the discussion:

- How have you experienced the program? What did you think of the program? *(The duration of 6 weeks, the timing for the sessions, the number of workshops, the location, the topics covered)*
- What feelings did you have? (*Identity, relationship to the others and to the body, new experiences, orientation to the future*) Could you tell me what you liked about the sessions? If anything bothered you?
- What have the sessions changed for you? *(Prompt with aspects of daily life, relations with those close, relations with caregivers?)*
- How come you stayed until the end of the 6 sessions? *(Or how come you didn’t stay until the end of the 6 sessions?)* If you were able to change something to improve the program, what would it be?
- Would you recommend the program to someone you care about? Why? What would you say to that person to advise them to take the program?
- That’s the end of the interview, is there anything you’d like to add?
